# Global feasible path planning for pest monitoring robots in unstructured agricultural environments

**DOI:** 10.3389/fpls.2026.1784030

**Published:** 2026-03-18

**Authors:** Yipeng Shao, Fazhan Tao, Pengju Si, Baofeng Ji, Mengyang Li

**Affiliations:** 1 Longmen Laboratory, Luoyang, China; 2 College of Information Engineering and Artificial Intelligence, Henan University of Science and Technology, Luoyang, China; 3 School of Electrical Engineering and Automation, Luoyang Normal University, Luoyang, China

**Keywords:** artificial potential field, cubic B-spline curve, dynamic goal-oriented probability sampling, rapidly-exploring random tree, sustainable agriculture, variable step size sampling

## Abstract

In the context of sustainable agriculture and precision agriculture, autonomous mobile robots play a pivotal role in intelligent plant pest and disease detection. However, navigation within complex agricultural environments—characterized by narrow crop rows and irregular obstacles—remains a persistent challenge. To address these limitations, this study presents a Goal-Oriented Adaptive Bidirectional RRT* (GABi-RRT*) algorithm designed to overcome the issues of low sampling efficiency and unstable path quality that are inherent in the conventional RRT* algorithm in narrow spaces. The proposed GABi-RRT* algorithm introduces several key innovations to improve its performance in complex environments. In the sampling phase, an innovative dynamic goal-oriented probabilistic sampling strategy is introduced. This strategy adaptively adjusts the sampling probability throughout the planning process, taking into account the distribution of obstacles between the current node and the target. This approach significantly enhances the efficiency of the sampling process compared to traditional random sampling methods. In the RRT* expansion phase, the algorithm integrates the RRT* expansion mechanism with an enhanced Artificial Potential Field (APF) and introduces an adaptive step-size strategy. This combination improves the algorithm's search efficiency and enhances its exploratory capabilities, allowing for better navigation in diverse and challenging environments. For path optimization, the algorithm employs a hybrid approach that combines pruning optimization with cubic B-spline curve fitting. This method eliminates redundant nodes and smooths the generated path, thereby improving path quality and reducing the turning frequency of the monitoring robots. Finally, comparative experiments in a continuous narrow environment simulating crop rows reveal that the proposed GABi-RRT* algorithm outperforms several existing algorithms, including Bias-RRT*, Informed-RRT*, and P-RRT*. Specifically, the GABi-RRT* algorithm reduces the average running time by 45.39%, 49.71%, and 71.52%, respectively, and shortens the path length by 5.70%, 3.97%, and 1.60%. These results demonstrate the superior capabilities of the GABi-RRT* algorithm in terms of path quality, stability, and search efficiency, making it a promising solution for autonomous navigation in agricultural environments.

## 1 Introduction

Driven by the rapid development of information technology, the traditional agricultural sector is undergoing a rapid transformation toward smart agriculture Mrutyunjay et al. (2024). Currently, a wide range of autonomous robots—from electric tracked robots to crop inspection platforms—are increasingly undertaking labor-intensive tasks such as crop scouting and spraying Huiping et al. (2024). However, path planning for robots in high-density greenhouses and plantations remains a challenging endeavor. In agricultural settings, greenhouse structures and tree canopies frequently obstruct satellite positioning signals, rendering satellite-based positioning systems unstable. Accordingly, to ensure the real-time responsiveness of robot planning, path planning algorithms must exhibit low computational complexity. Concurrently, under the premise of maintaining low algorithmic computational overhead, the efficiency and quality of path planning are equally critical, as they directly impact the overall operation Yuchao et al. (2024).

Agricultural navigation strategies can be classified into three categories: graph-based search methods, intelligent optimization algorithms, and sampling-based algorithms. Firstly, traditional heuristic approaches and Voronoi sampling-driven approaches Chatzisavvas et al. (2024); Rasekhipour et al. (2018); Tahilyani et al. (2022) exhibit reliability in structured environments; however, their computational complexity escalates drastically in large-scale spaces with complex obstacles, primarily due to the curse of dimensionality. The second class encompasses intelligent planning algorithms, including reinforcement learning Roque-Claros et al. (2024); Mao et al. (2025); Sharma et al. (2020), ant colony optimization, and genetic algorithms Fan (2023); Liu et al. (2022); Zheng et al. (2023). While these algorithms demonstrate strong environmental adaptability, they demand extensive training datasets or meticulous parameter tuning. The third category includes algorithms based on sampling, such as the Probabilistic Roadmap (PRM) Ma et al. (2022), which excel in the efficient exploration of high-dimensional configuration spaces. Notably, the model-free properties of the Rapidly-exploring Random Tree (RRT) algorithm LaValle (1998) and its improved version, RRT* Karaman and Frazzoli (2011) render them particularly suitable for unstructured environments. Nevertheless, their practical deployment on agricultural robots remains challenging.

Modern high-density planting environments resemble topological mazes, where narrow corridors, erratic canopy growth, and irregular infrastructure frequently occlude the robot’s perception Ye et al. (2023). The standard RRT* algorithm underperforms in such scenarios: its random sampling mechanism hinders efficient exploration of narrow passages, leading to suboptimal computational efficiency Kan (2024). More critically, the stochastic sampling paradigm often generates tortuous, oscillatory paths. For robots equipped with high-resolution cameras, this “zigzag” motion profile is infeasible, as it induces mechanical vibrations and motion blur, compromising the reliability of plant detection. Furthermore, robots must not only avoid static obstacles but also minimize encroachment on crops themselves—a nuance unaddressed by conventional global planners Mengqi and Hanxi (2023). Only by ensuring path smoothness and safety can a stable global guidance trajectory be established to support subsequent dynamic operations.

To address the shortcomings of the RRT* planning algorithm in smart agricultural environments, multiple studies have proposed targeted improvement strategies in recent years. Given the complexity of terrain perception in orchards, environmental mapping forms the fundamental basis for path planning. Cao et al. (2025) proposed a framework rooted in traversability analysis. This framework integrates an enhanced LeGO-LOAM algorithm for high-precision mapping and employs the RRT* algorithm to generate paths within the constructed point cloud map, effectively addressing the failure of standard algorithms on muddy or uneven terrain. However, the path planning module in this work is merely a post-mapping step and does not specifically tackle redundant nodes to minimize steering maneuvers, which would otherwise reduce control energy consumption on soft ground. To boost algorithmic search efficiency, Zhang et al. (2024) adopted a dual-grid map hierarchical strategy. By stratifying the processing of static and dynamic obstacles, this approach significantly accelerates the computational speed of the RRT algorithm in complex orchard settings. Nevertheless, grid discretization inherently limits the resolution of the generated paths. In the context of coverage requirements for spraying robots, Lian et al. (2024) presents tailored modifications. However, the resulting paths still exhibit abrupt turns, and excessive steering is detrimental to the implementation of precision agriculture. Regarding the mechanical motion constraints of robots, Hu et al. (2025) introduced the HPS-RRT* algorithm. This algorithm enhances sampling efficiency by integrating a novel heuristic guidance strategy and a path-smoothing mechanism fused with the Sparrow Search Algorithm (SSA). Specifically, the SSA enables dynamic curvature adjustment, which not only ensures strict adherence to nonholonomic constraints but also significantly improves path smoothness. However, the incorporation of the SSA introduces computational overhead: since the SSA requires multiple iterations to converge, integrating this mechanism into the post-processing phase may prolong the overall planning time. Ye et al. (2023) adopts a bidirectional RRT strategy and reinforces kinematic constraints, leading to a marked reduction in algorithmic search time. However, the connection points between the two trees often result in sharp turning angles, leaving room for improvement in minimizing steering amplitude and frequency. Recent research on the improvement of RRT* for agricultural robotic arms and unmanned aerial vehicles (UAVs) mainly focuses on high-dimensional planning and algorithm integration. Liu et al. (2024) utilized bidirectional RRT combined with RGB-D information to address the local extremum problem of a 6-DOF picking arm, but the redundant nodes generated by this method still require pruning mechanisms to enhance path smoothness. Castro et al. (2023) proposed a hierarchical framework integrating RRT and deep reinforcement learning (DRL) for monitoring UAVs, with RRT and DRL respectively responsible for global search and local obstacle avoidance. Although this hybrid strategy is a current research hotspot, the high computational and learning costs brought by DRL limit its application in low-cost agricultural robot systems.

Beyond the domain of smart agriculture, numerous valuable strategies and methodologies for RRT* improvement have been proposed in other application scenarios. Jeong et al. (2019) introduced the Q-RRT* algorithm, which expands the search radius for parent node selection to reduce path costs while preserving asymptotic optimality. Liao et al. (2021) proposed F-RRT*, an alternative approach that employs binary search to identify distant parent nodes instead of confining the search to nodes within the reconnection hypersphere. This strategy accelerates the convergence of the tree toward higher-level parent nodes; however, since the sampling process of both Q-RRT* and F-RRT* remains stochastic, the quality of the paths they generate is still largely dependent on the quality of their random sampling. In response to this limitation, scholars have started investigating integrated algorithmic approaches to capitalize on the synergistic advantages of various techniques. P-RRT* Qureshi and Ayaz (2016) integrates the Artificial Potential Field (APF) with RRT*, while PQ-RRT* Li et al. (2020) and PF-RRT* Fan et al. (2022) combine the APF-guided tree growth mechanism of P-RRT* with the optimality properties of Q-RRT* and F-RRT*, respectively. Nevertheless, integration challenges persist: specifically, when the start or goal regions are densely cluttered with obstacles, the guidance from the APF potential field tends to trap the algorithm in local minima, resulting in stagnation. Feng et al. (2024) addressed this issue by proposing DBVSB-P-RRT*, an algorithm well-suited for rapid path initialization in cluttered start environments. However, it is not without drawbacks—it struggles to navigate narrow passages effectively, and the quality of the paths it generates remains inconsistent.

Despite extensive research efforts dedicated to the enhancement of the RRT* algorithm, challenges still persist regarding the environmental adaptability, computational efficiency, and smoothness of generated paths for improved RRT* variants. This study presents the GABi-RRT* algorithm., which integrates comprehensive optimization strategies across the sampling, expansion, node connection, and path optimization phases. The main contributions of this work are summarized below:

1. An adaptive target deviation probability sampling strategy for agricultural environments is proposed in this paper, where the deviation probability is regulated by the number of obstacles between the present node and the goal node. This method optimizes the generation of random samples by dynamically adjusting the target-oriented sampling mechanism. Therefore, it mitigates the inherent randomness of the sampling process while enhancing exploratory attention to the target area.
2. We propose a strategy that tightly integrates bidirectional RRT* with the artificial potential field (APF) technique. Based on the APF framework, an adaptive attractive function is introduced to address common issues such as local optima and unreachable goals. An extended heuristic algorithm with dynamic step size is introduced. This approach aims to achieve a delicate balance, improve path quality, And ensure its adaptability to the environmental conditions of complex vegetation and ridged field systems.
3. This paper enhances path quality by integrating pruning optimization with cubic B-spline fitting. The pruning strategy, rooted in the triangle inequality, eliminates redundant nodes to boost efficiency. Concurrently, cubic B-spline fitting smooths abrupt turns and reduces steering frequency. Crucially, this smoother motion profile minimizes mechanical vibration and camera jitter, mitigating motion blur to ensure high-fidelity image acquisition for precise pest detection.

The organizational structure of the subsequent content of this article is arranged as follows: Section 2 delineates three canonical problems in path planning. Section 3 provides an overview of the foundational RRT*, Bi-RRT*, and Informed-RRT* algorithms. Subsequently, Section 4 elaborates on our proposed GABi-RRT* algorithm. Finally, Section 5 establishes various simulated environments to systematically assess the performance of path planning using the GABi-RRT* algorithm across scenarios of varying complexity. Through comparative experiments with several mainstream algorithms, the superiority of GABi-RRT* is proved. Section 6 concludes the paper and discusses limitations and future work.

## 2 Problem definition

Motion planning is formulated within the configuration space, *Q*. This space is partitioned into a disjoint obstacle space, *Q_obs_*, and a free space, *Q_free_*, such that *Q* = *Q_free_* ∪ *Q_obs_*. The objective is to find a continuous path, defined as a mapping path: [1, end] → *Q*, that connects a start configuration *q_start_* ∈ *Q_free_* to a goal configuration *q_goal_* ∈ *Q_free_*.* A* path is considered feasible or collision-free if it resides entirely within the free space: ∀*τ* ∈ [1, end], path(*τ*) ∈ *Q_free_*.

Definition 1 (Feasible Path). A path is deemed feasible if it represents a continuous, collision-free trajectory connecting *q*_start_ to *q*_goal_. It must satisfy the boundary conditions *path*(1) = *q*_start_ and *path*(end) = *q*_goal_. The inability to identify such a path constitutes a planning failure.

Definition 2 (Optimal Path). An optimal path is a member of the set of all feasible paths that minimizes a given cost function, such that *c*(path) = min{*c*(path): path ∈ feasible path}, where *c*(path) is the length of the path. A failure is reported if no feasible solution exists.

Definition 3 (Time-Optimal Planning). The goal of time-optimal planning is to find a feasible path while reducing the computational time consumed by the planning algorithm. The performance is measured by the planning duration, defined by the expression *time*(path) = min{*time*(path): path ∈ feasible path}, where *time*(path) represents the computational time required to generate the path.

## 3 Relate work

This section begins with a detailed exposition of the core principles underlying the RRT* algorithm. The RRT* algorithm serves as the foundational framework for the method proposed in this paper. Subsequently, we present an in-depth discussion of Informed RRT*. As a significant paradigm for heuristic enhancements to RRT*, Informed RRT* serves as a primary benchmark for evaluating the performance of the GABi-RRT* algorithm.

### 3.1 RRT*

The RRT* algorithm represents an advancement over standard RRT, distinguished by its integration of parent re-selection and tree rewiring mechanisms. Therefore, a thorough grasp of RRT* requires a preliminary review of the fundamental RRT algorithm. This algorithm is a probabilistically complete, sampling-based planner that iterates through four distinct stages: ampling, expansion, collision detection, and path connection. In every iteration, a random configuration *q_rand_* is drawn from the admissible configuration space. The existing tree, *T*, is then queried to identify the nearest node, *q_nearest_*. Next, a new node *q*_new_ is created through extension from *q_nearest_* is extended toward *q_rand_* using a predetermined step size. Finally, the path segment leading to this new node is checked for collisions. Upon successful collision detection, *q*_new_ is added to the tree structure *T*. This expansion loop repeats until a new node enters the goal region.

RRT* represents a milestone improvement over the RRT algorithm, achieving superior path quality through two core strategies: optimal parent selection and neighborhood rewiring. The detailed implementation of the algorithm is presented in **Algorithm 1**. It takes the initial configuration *q_start_*, goal configuration *q_goal_*, obstacle space *Q_obs_*, and iteration limit *Iter* as inputs to progressively construct a final tree graph, *G* = (*V, E*). In this equation *V* denotes the collection of all visited nodes, while *E* represents the set of edges forming the random tree.

Algorithm 1

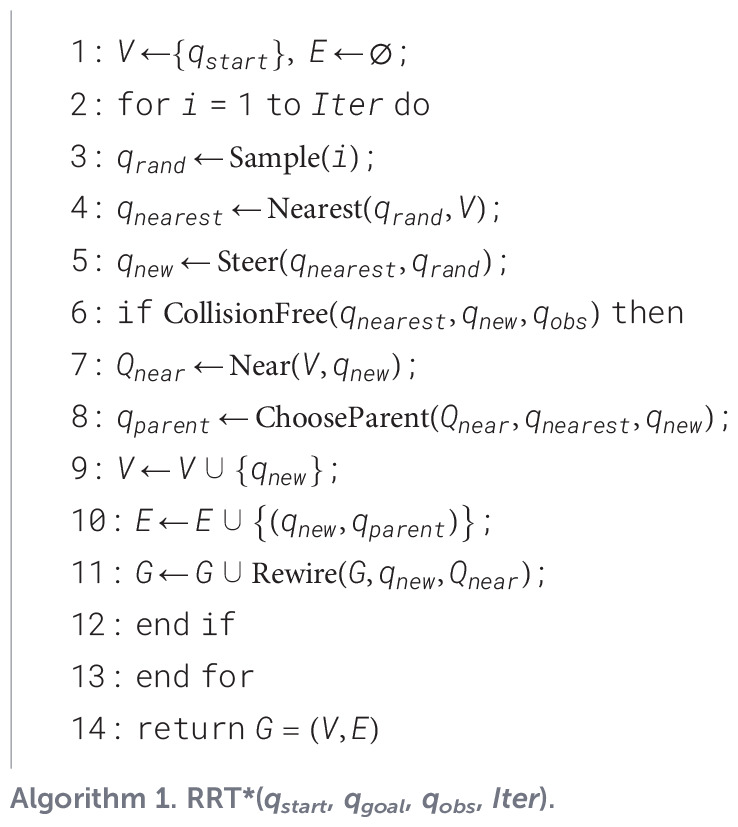

**Figure 1:**
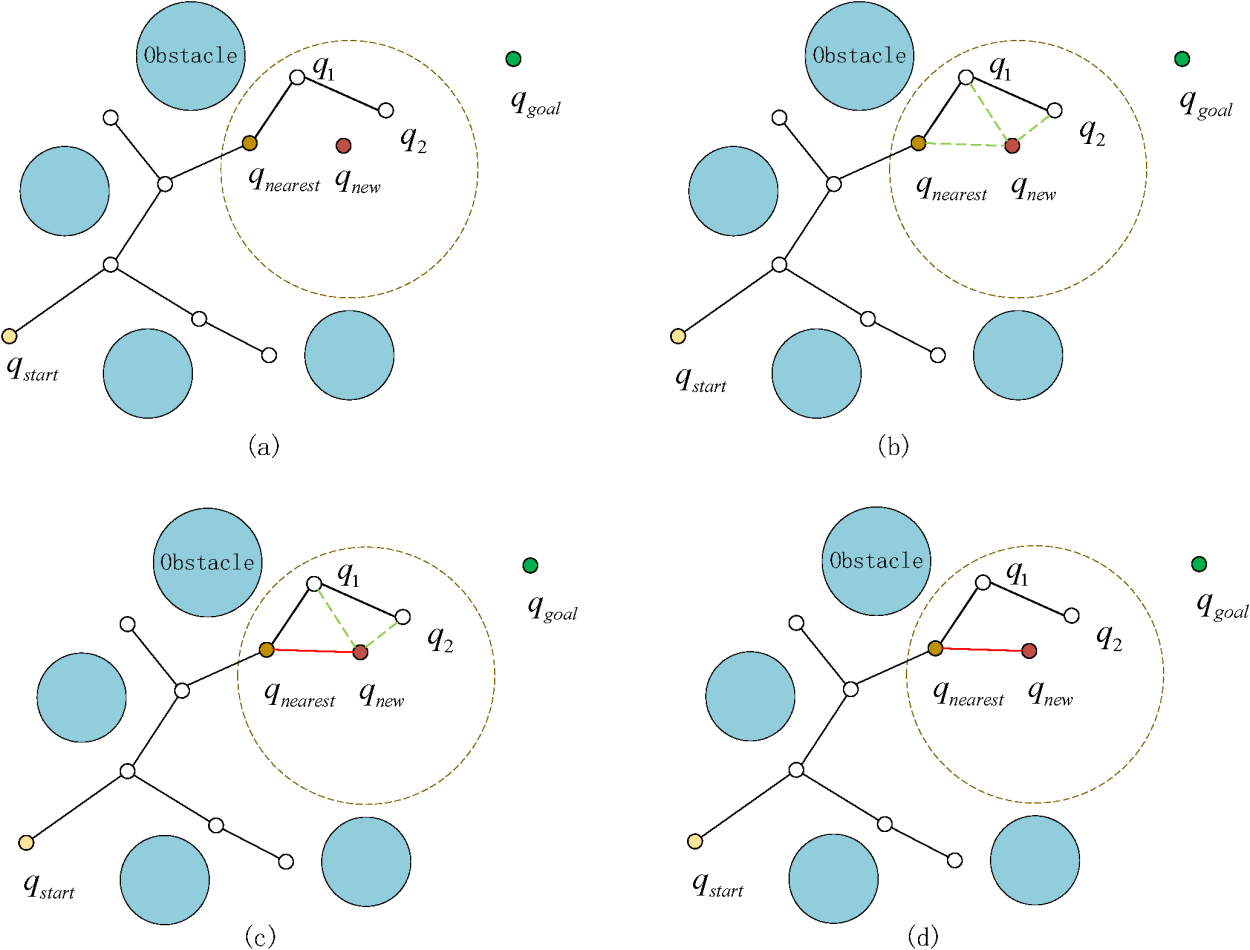
RRT* path reconnection diagram.

ChooseParent strategy implements the parent node re-selection process, as illustrated in **Figure 1**. The algorithm first searches for all reachable nodes within the neighborhood of *q_new_*. Then, the path cost from the initial point *q_start_* to *q_new_* through each candidate node is computed using a cost function. The candidate node yielding the minimum cost is then chosen as the optimal parent of *q_new_*. Rewire strategy performs the rewiring operation. It treats *q_new_* as a potential new parent node and evaluates the path costs to the existing nodes within its neighborhood. If a lower-cost path to any of these neighboring nodes is found via *q_new_*, the parent of that node is updated to *q_new_*.

These two optimization algorithms together form the mechanism that guarantees the asymptotic optimality of RRT*, enhanced the algorithm’s rate of convergence. However, this optimization increases the computational complexity, each iteration requires additional neighborhood queries, cost evaluations, and tree structure adjustments. Consequently, the RRT* algorithm requires considerably more time on average to complete a search than the basic RRT, a difference that is particularly pronounced in complex environments. Therefore, a central challenge in the practical application of RRT* is the need to strike an appropriate balance between the conflicting requirements between computational performance and the quality of the resulting path.

### 3.2 Informed RRT*

**Figure 2:**
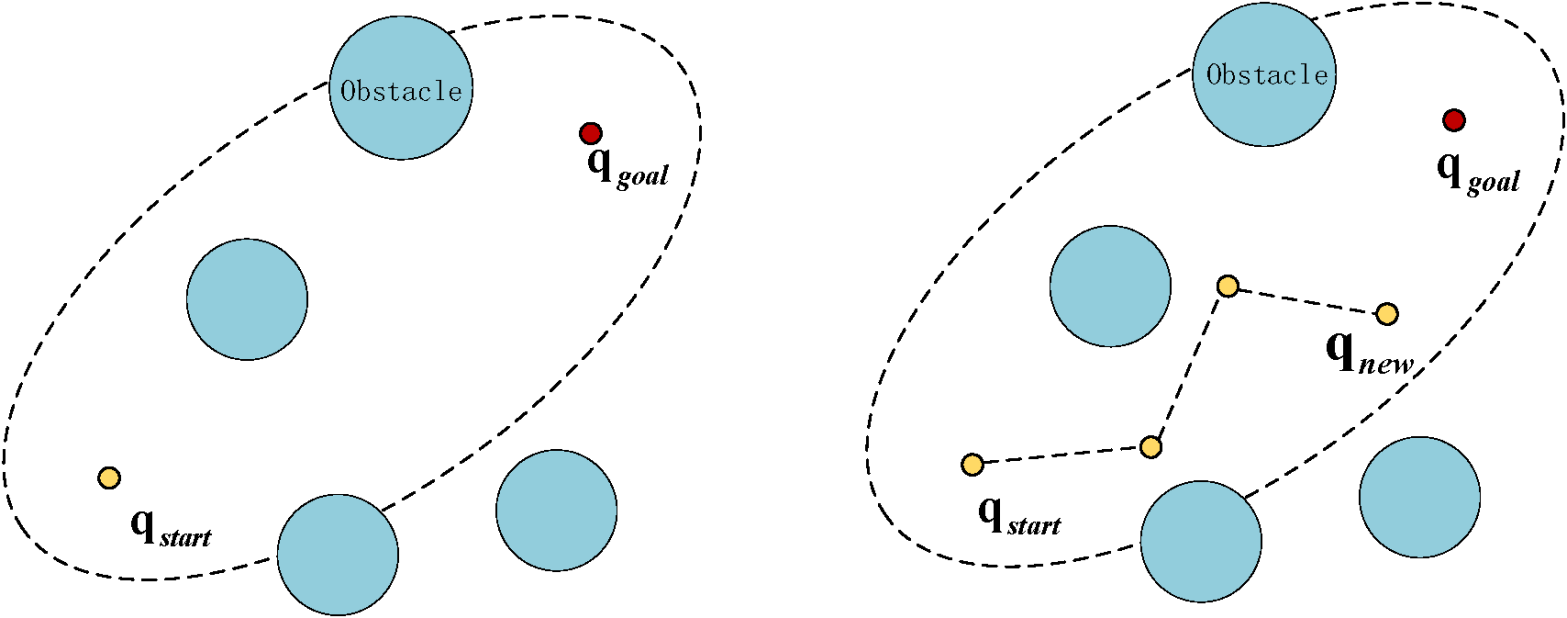
Informed-RRT*.

The Informed RRT*, an enhanced variant of the Rapidly-exploring Random Tree Star (RRT*), addresses path planning challenges by introducing an ellipsoidal heuristic to focus sampling within a dynamically defined region after an initial solution is found, significantly improving convergence toward an optimal path. Compared to RRT*, its primary advantage lies in its faster convergence rate. The algorithm constrains the sampling region to an ellipsoid that encompasses the optimal path. This strategy eliminates redundant exploration in other regions, as illustrated in **Figure 2**. This heuristic approach not only accelerates convergence but also maintains robust search capabilities, enhancing computational efficiency while preserving asymptotic optimality. Additionally, it retains RRT’s ability to handle complex, non-convex environments and avoids the local minima pitfalls of gradient-based methods. However, the limitations of this algorithm are also notably apparent; specifically, its performance is heavily contingent upon the quality of the path solution. A suboptimal initial path can lead to an inflated ellipsoid volume, resulting in inconsistent convergence rates across multiple trials. Furthermore, like other sampling-based methods, it struggles with narrow passages due to reliance on random sampling, and its deterministic rejection of samples outside the ellipsoid may occasionally exclude regions critical for further optimization. In summary, the Informed RRT* algorithm strikes a favorable balance between optimality and efficiency in static, high-dimensional problems. However, it remains constrained by its reliance on the variable quality of the initial solution and the persistent issue of relatively blind sampling.

## 4 Goal-oriented adaptive bidirectional RRT*

Building upon the analysis of RRT* and its variants (specifically Informed-RRT*), this section introduces the proposed GABi-RRT* algorithm. This algorithm implements comprehensive improvements regarding the sampling stage, extension, and path optimization phases of RRT*. First, regarding the sampling stage, we designed a novel goal-oriented probabilistic mechanism. Based on an evaluation of obstacle density between a new node and the goal, this mechanism enables the algorithm to adaptively modify the goal-directed sampling probability, thereby enhancing both the quality of the initial path and obstacle avoidance capabilities. During the extension process, the algorithm introduces a novel attraction function (inspired by P-RRT*) alongside an adaptive step-size strategy. This function enhances the algorithm’s flexibility in diverse environments and boosts search efficiency, effectively reducing undirected exploration while elevating the general quality of the route. Finally, the generated path undergoes optimization via a two-step post-processing procedure: a pruning method based on the triangle inequality and a smoothing technique utilizing B-splines. The subsequent subsections will expound upon these strategies in detail. **Algorithm 2** outlines the complete execution workflow of the GABi-RRT* algorithm.

Algorithm 2

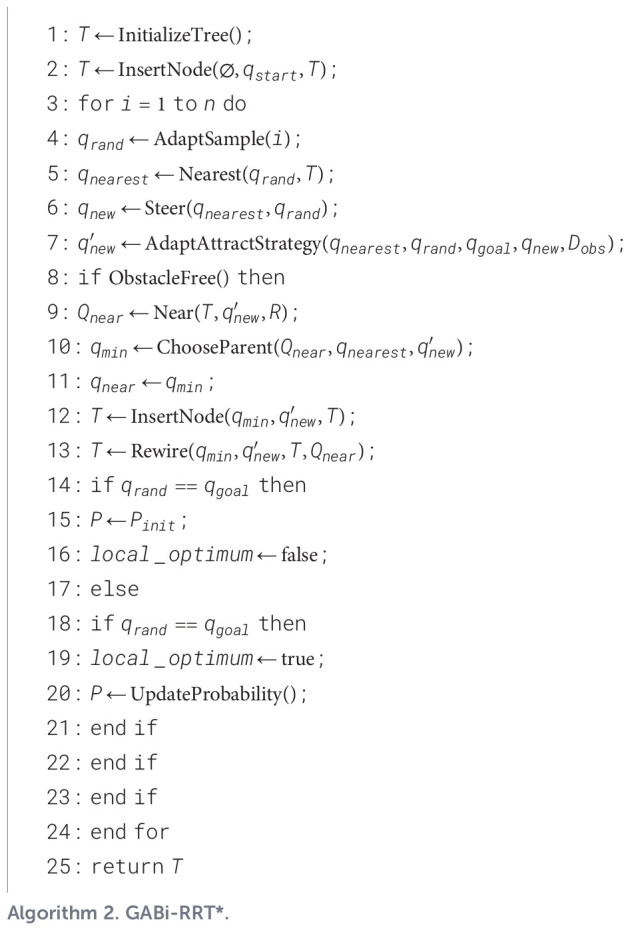

Among them, *AdaptAttractStrategy*() is the adaptive attraction strategy. The pseudo-code is shown as **Algorithm 3**. *AdaptSample*() is the variable probability sampling strategy. The fundamental concept behind this mechanism is to monitor the convergence state of the algorithm in real time and automatically adjust the value of the probability parameter P when the local optimal situation is detected, thereby effectively maintaining a trade-off between the algorithm’s stochastic exploration and its guided directional search abilities. For detailed implementation steps and parameter update rules, please refer to the pseudo-code description in **Algorithm 4**.

Algorithm 3

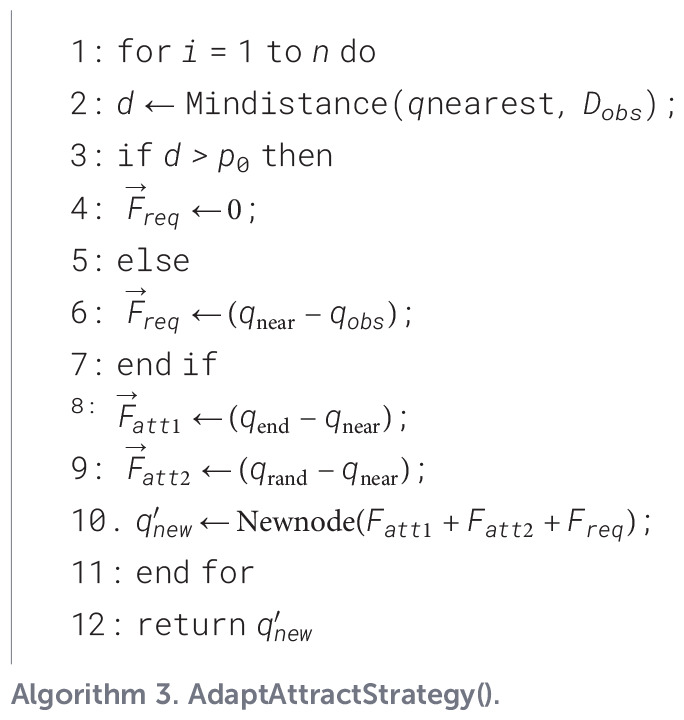

Algorithm 4

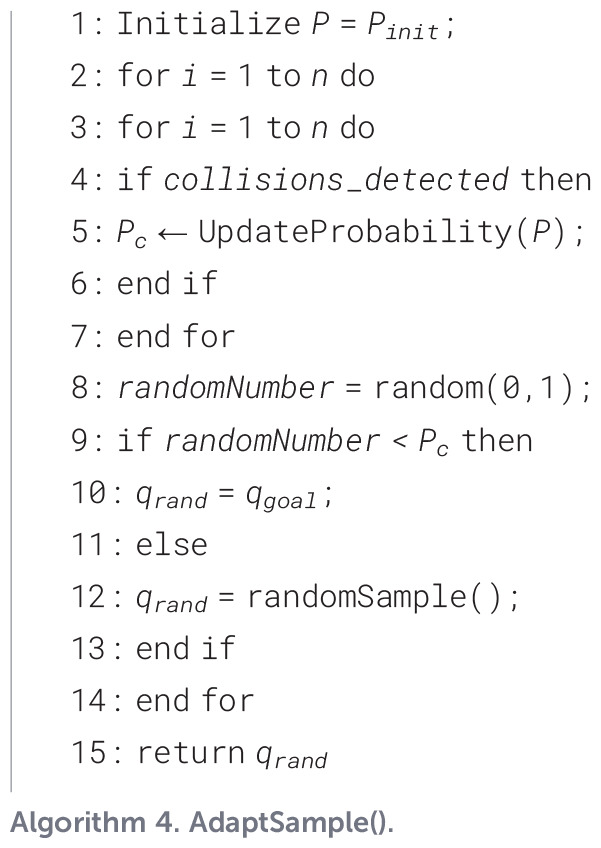

### 4.1 Bidirectional target bias sampling strategy

**Figure 3:**
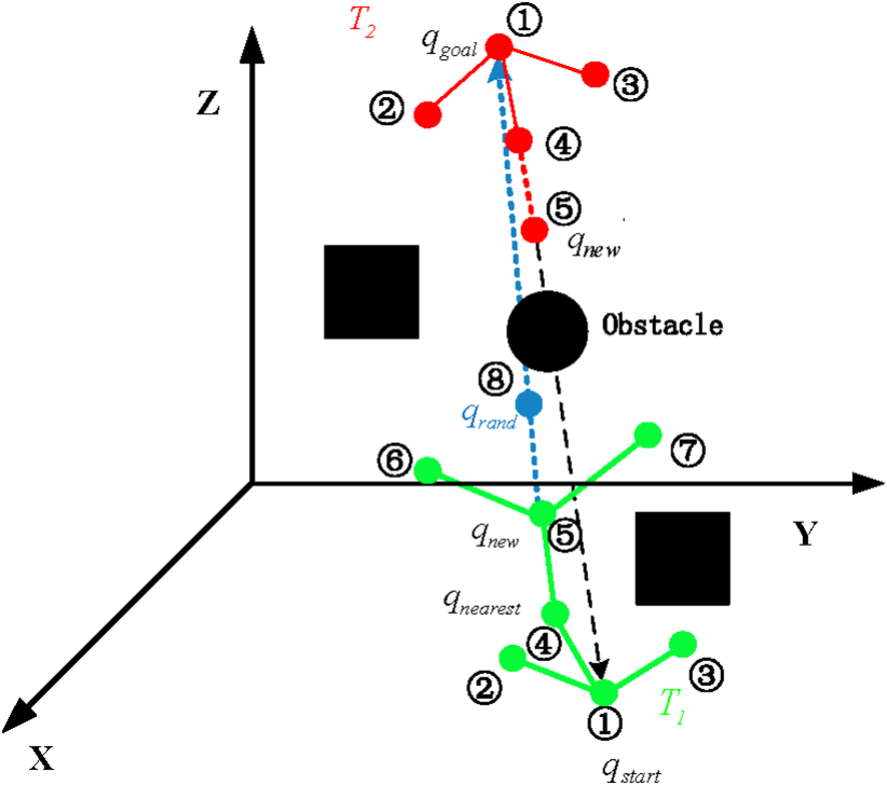
Bidirectional-RRT* with goal-bias sampling.

The RRT* algorithm explores the unexplored areas by randomly sampling throughout the entire space. It reaches the destination by connecting the sampling points and generates the initial path. However, pure random sampling is inherently blind, thereby reducing the algorithm’s effectiveness in navigating complex environments and unstable quality of the generated initial path. To address this, the guidance strategy for the two trees (Tree1 and Tree2) was redesigned with the introduction of a bidirectional search mechanism, along with adjustments to the selection of guiding points in the goal-biased probability. Unlike a single random tree that directly selects the goal point as the guiding point, the guiding points for the two trees in bidirectional search need to be independent and appropriately chosen to optimize search efficiency. In this study, the initial position and target location serve as reference guidance points, Tree1 uses the goal point as its guiding point, while Tree2 uses the start point as its guiding point. As shown in **Figure 3**, the new node ❹ in Tree1 is generated from node ❸ of Tree1 using the goal point as a guiding reference; similarly, the new node ❸ in Tree2 is generated from node ❹ of Tree2 using the start point as a guiding reference. By independently guiding the two trees with the goal and start points respectively, the bidirectional search achieves both parallelism and high efficiency, with each tree growing in a direction uninfluenced by the other.

This guide point selection method can effectively coordinate the growth directions of two random trees, thereby improving the search efficiency. The probabilistic target bias sampling used in the sampling process is:

(1) $$q_{\text{rand}\;}=\{\begin{array}{ll} \;\text{Random},\; & P_{r}>P_{c}\\ q_{\text{goal}\;}\;\text{or}\;q_{\text{start}\;}, & \;\text{else}\; \end{array}$$

where, *P_λ_* is the random probability value of the sampling, and *P_c_* is the set sampling probability. Since the improved algorithm is a bidirectional search, the target point can also be *q_goal_* or *q_start_*. The sampling probability is expressed as:

(2) $$P_{c}=0.1+0.9e^{-\frac{{\left(C_{n}+1\right)}^{2}}{2}}$$

where, *C_n_* is a positive integer and represents the quantity of collisions with obstacles. With the increase of *C_n_*, the value *P_c_* decreases accordingly. In other words, during the sampling process, if the algorithm encounters a region with numerous or large obstacles, the expansion of the random tree toward the goal is restricted. The very attraction from the goal, which is intended to guide the search, becomes counterproductive. Its influence diminishes as it continuously pulls the tree toward an impassable area, thereby hindering the exploration of alternative, viable paths. The quantity of randomly sampled points ought to be raised. Conversely, if the number of collisions *C_n_* is smaller, *P_c_* should increase, and the randomly generated tree should expand the goal point as a random sampling node.

**Figure 4:**
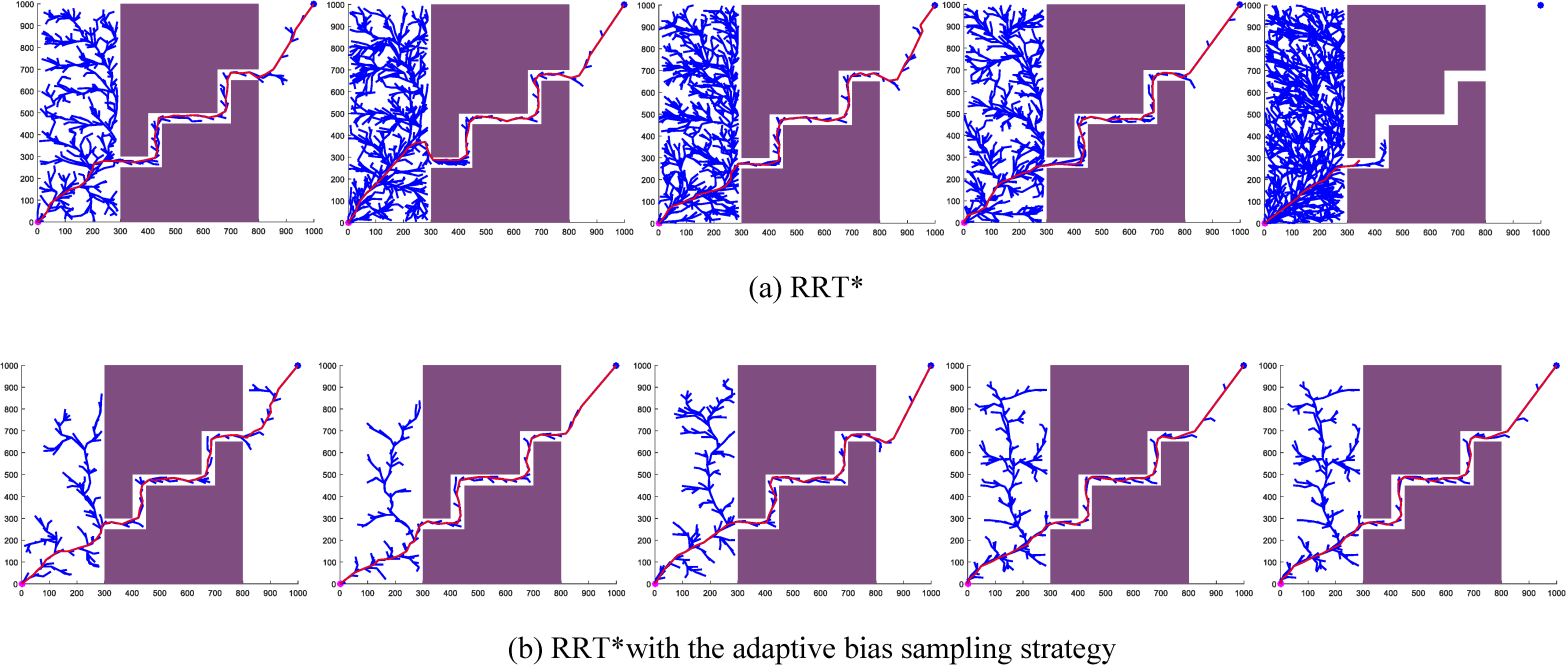
RRT* with goal-bias sampling.

We introduces a dynamic goal-biased sampling strategy whereby the probability of sampling the goal region progressively increases as the search advances; this approach serves to accelerate convergence toward the target while simultaneously enhancing the optimality of the generated path. To evaluate the performance of this adaptive biasing approach, we conducted five sets of comparative experiments benchmarking the standard RRT* algorithm against our proposed approach. The results obtained from 3,000 sampling iterations within a continuous narrow scenario are illustrated in **Figure 4**.

The results demonstrate a significant performance degradation of he conventional RRT* algorithm in environments with narrow passages. This deficiency, manifesting as slow convergence and occasional planning failures, stems from its fixed target-biasing strategy. The fixed bias leads to excessive sampling in these constrained regions, hindering broader exploration and trapping the algorithm in local minima. In contrast, our proposed RRT* variant with an adaptive biasing strategy dynamically adjusts the sampling focus. This allows the algorithm to navigate narrow channels more efficiently, effectively mitigating the issue of premature convergence observed in the standard RRT*.

### 4.2 Adaptive attraction strategy

The P-RRT* algorithm represents a synergistic fusion of the Artificial Potential Field (APF) and RRT* methods, leveraging the potential field concept to guide the search process. This integration serves a dual purpose: the repulsive potential emanating from obstacles steers the tree’s expansion away from high-risk regions, thereby enhancing collision-avoidance, while the attractive potential from the goal biases the search, accelerating convergence. The foundational principles of the APF, which P-RRT* adapts, are defined by the following potential functions:

(3) $$U_{att}(x)=\frac{1}{2}k_{a}\rho_{g}^{2}(x)$$

(4) $$U_{rep}(x)=\{\begin{array}{ll} \frac{1}{2}k_{r}{\left(\frac{1}{\rho (q,q_{obc})}-\frac{1}{\rho_{0}}\right)}^{2} & \rho (x)\leq \rho_{0}\\ 0 & \rho (x)>\rho_{0} \end{array}$$

Among them, the gain coefficients of the gravitational field and the repulsive field are represented by *k_a_* and *k_r_*, respectively. The vector $\rho_{g}(x)$ denotes the directional displacement from a given point *x* to the target location $q_{\text{goal}}$, and its norm, $\left\|\rho_{g}(x)\right\|$, corresponds to the Euclidean distance separating these two points.

The classic Artificial Potential Field (APF) algorithm steers a agricultural robot towards its destination by establishing a composite potential field. This field results from the combination of the attractive potential created by the goal and repulsive potentials produced by surrounding obstacles. The overall potential field is expressed as:

(5) $$U_{total}=\sum U_{att}+\sum_{i=1}^{n}U_{rep}$$

The intensity of the gravitational field and repulsive force is defined by the negative gradient of their respective potential functions.

(6) $$F_{att}(x)=k_{a}\rho_{g}(x)$$

(7) $$F_{rep}(x)=\{\begin{array}{ll} k_{r}{\left(\frac{1}{\rho (x)}-\frac{1}{\rho_{0}}\right)}^{2}\frac{1}{\rho^{2}(x)}\nabla \rho (x) & \rho (x)\leq \rho_{0}\\ 0 & \rho (x)>\rho_{0} \end{array}$$

The total force acting on the agricultural robot is:

(8) $$F_{att}(x)=\sum F_{att}+\sum F_{rep}$$

In response to the problems related to local optima and failure to achieve the goal, we modified the obstacle repulsion force field model employed in the traditional artificial potential field approach, and added an adjustment factor $\rho^{n}(x_{n},x_{goal})$. This guarantees that both the repulsive force and gravitational effect diminish to zero only upon the agricultural robot’s arrival at the target location. Therefore, the improved APF function added to the RRT* algorithm in this paper is as follows:

(9) $$U_{att}(x)=\{\begin{array}{ll} \frac{1}{2}k_{a}\rho^{2}(x_{n},x_{\text{go}al}) & \rho (x_{n},x_{\text{go}al})\leq d_{\text{go}al}^{*}\\ d_{\text{go}al}^{*}k_{a}\rho (x_{n},x_{\text{g}\circ al})-\frac{1}{2}k_{a}{\left(d_{\text{go}al}^{*}\right)}^{2} & \rho (x_{n},x_{\text{go}al})>d_{\text{go}al}^{*} \end{array}$$

(10) $$U_{rep}(x)=\{\begin{array}{ll} \frac{1}{2}k_{r}{\left(\frac{1}{\rho (x_{n},x_{\text{obs}})}-\frac{1}{\rho_{0}}\right)}^{2}\rho^{n}(x_{n},x_{\text{goal}}) & \rho (x_{n},x_{\text{obs}})\leq \rho_{0}\\ 0 & \rho (x_{n},x_{\text{obs}})>\rho_{0} \end{array}$$

where $x_{n},x_{goal}$ and $x_{obs},$ represent $q_{nearest}$, $q_{goal}$ and the obstacle’s location, respectively. $\rho (x_{n},x_{obs})$ denotes the Euclidean distance between $q_{nearest}$ and the closest obstacle, $\rho (x_{n},x_{goal})$ is the Euclideandistance from $q_{nearest}$ to $q_{goal}$, and M is an arbitrary constant.

The attractive force acting on the robot is defined by the negative gradient of the attractive potential.

(11) $$F_{att}(x)=-\nabla U_{att}(x)=\{\begin{array}{ll} k_{a}\rho (x_{n},x_{goal}) & \rho (x_{n},x_{goal})\leq d_{goal}^{*}\\ \frac{d_{goal}^{*}k_{a}\rho (x_{n},x_{goal})}{\rho (x_{n},x_{goal})} & \rho (x_{n},x_{goal})>d_{goal}^{*} \end{array}$$

The potential energy function of this attraction is defined in segments, and its performance depends on a threshold distance $d_{goal}^{*}$. For configurations whose distance from the target $\rho (x_{n},x_{goal})$ falls within this threshold range $\rho (x_{n},x_{goal})\leq d_{goal}^{*}$, the potential energy magnitude is directly proportional to the squared distance, ensuring that the agricultural robot has sufficient attraction when approaching the target. Conversely, when $\rho (x_{n},x_{goal})>d_{goal}^{*}$, the magnitude of the potential energy will decrease. This design choice helps to avoid the planner getting trapped in a local minimum due to overly strong attraction when dealing with long distances.

During the process of the agricultural robot moving towards the goal point, both the attractive and repulsive forces acting on it experiences will decrease to a certain extent. Only when the agricultural robot reaches the target point will influence both the gravitational force and the repulsive force simultaneously decrease to zero, that is to say, the target point becomes the point with the minimum potential energy. This can solve The inherent drawback of premature convergence and the inability of the traditional P-RRT algorithm to achieve the goal. When the agricultural robot has not reached the goal point, the corresponding repulsive force function is:

(12) $$F_{rep}(x)=-\nabla U_{rep}(x)=\{\begin{array}{cc} F_{rep1}n_{1}+F_{rep2}n_{2} & \rho (x,x_{obs})\leq \rho_{0}\\ 0 & \rho (x,x_{obs})>\rho_{0} \end{array}$$

(13) $$\left\{ \begin{array}{l} F_{rep1}=k_{r}(\frac{1}{\rho (x_{n},x_{obs})}-\frac{1}{\rho_{0}})\frac{\rho^{n}(x_{n},x_{goal})}{\rho^{2}(x_{n},x_{obs})}\\ F_{rep2}=\frac{n}{2}k_{r}{\left(\frac{1}{\rho (x_{n},x_{obs})}-\frac{1}{\rho_{0}}\right)}^{2}\rho^{n-1}(x_{n},x_{goal}) \end{array}\right.$$

where $F_{req1}$ is aligned with the direction of $-n_{1}$ to point towards the obstacle, for $n_{1}=\nabla \rho (x,x_{obs})$, and $F_{req2}$ is aligned with $n_{2}=-\nabla \rho (x,x_{goal})$ to point towards the target.

During each expansion process, the adaptive attraction function dynamically adjusts the expansion direction using the Euclidean distance from the node in the tree to the goal as the determining factor. When the distance is far, this function will more strongly guide the tree node to grow in the direction of the target. While when the distance is relatively close, it allows for more random expansions to explore the local path.

**Figure 5:**
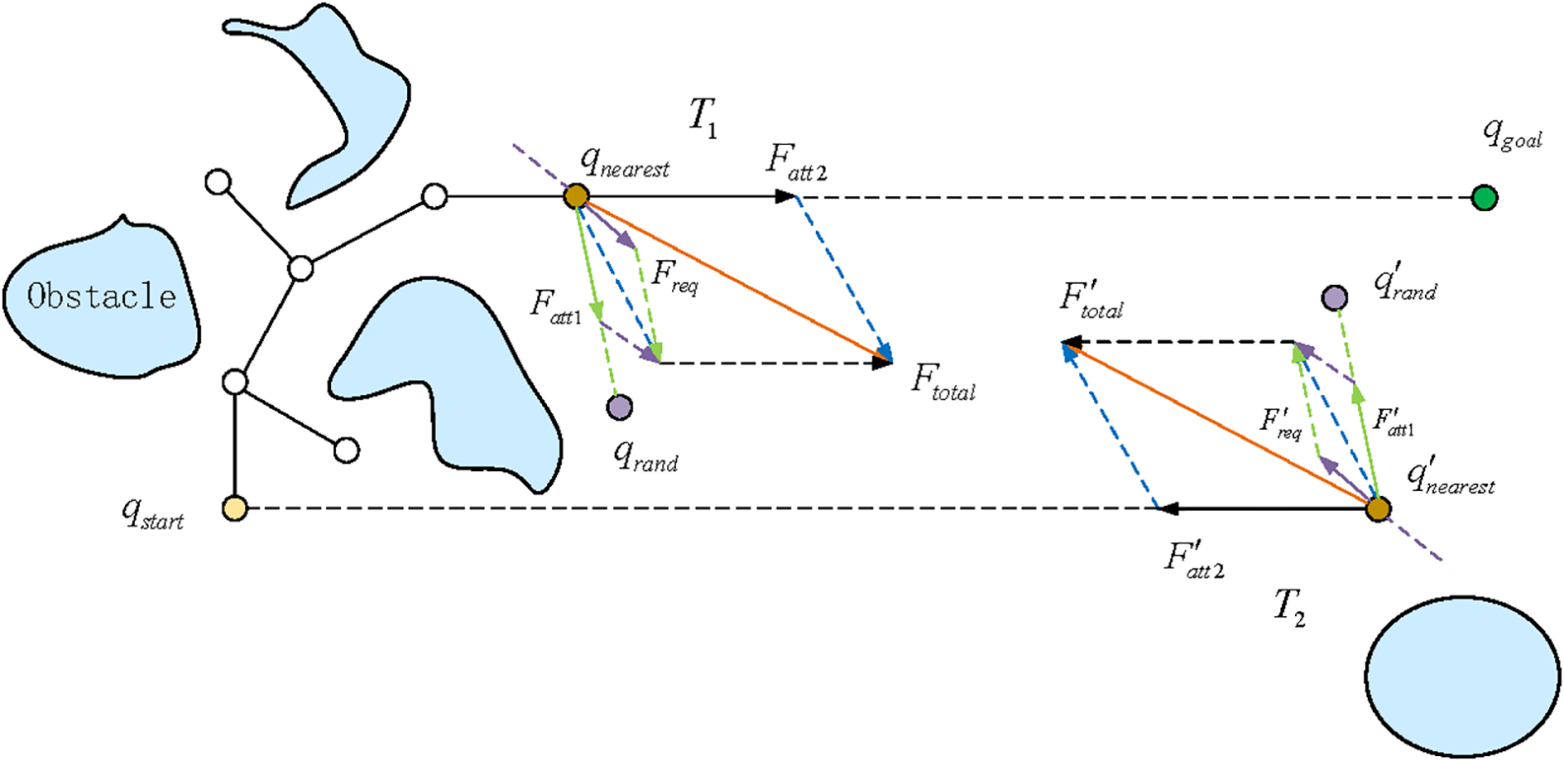
Analytical diagram of *q_nearest_* force of bidirectional RRT* in enhanced potential field.

As depicted in **Figure 5**, the extension from the nearest node, *q*_nearest_, towards a new node, *q*_new_, is governed by a composite force. This force comprises two attractive components, *F*_att1_ from the target and *F*_att2_ from a random sample, and a repulsive component, *F*_rep_, from obstacles. The resultant of these potential field force vectors dictates the current direction of expansion, with the new node *q*_new_ being positioned along this vector at a distance defined by the adaptive step size. This generation strategy, guided by the improved force mechanism, effectively enhances the algorithm’s exploration capabilities.

### 4.3 Adaptive step size expansion

In improved algorithms based on RRT*, environmental complexity fundamentally influences the optimal magnitude of the current expansion step. In environments with sparse obstacles, a larger step size serves to reduce the total number of required nodes, thereby boosting search efficiency. Conversely, within narrow passages or cluttered environments, the search efficiency of RRT* algorithms employing larger step sizes typically deteriorates, making a smaller step size critical for effective exploration. Consequently, drawn from both theoretical derivation and extensive experimentation, our conclusion is that the optimal step size is inversely proportional to obstacle density. To implement a dynamic step size, we adopt an adaptive strategy that relies on two novel metrics to quantify local environmental clutter: the dynamic obstacle count ratio, $B_{n}$, and the obstacle space area ratio, $B_{a}$. The calculation methods for these metrics are defined as follows:

(14) $$B_{n}=\frac{n_{obs}}{S_{total}}$$

(15) $$B_{s}=\frac{s_{obs}}{S_{total}}$$

where, $n_{obs}$ and $s_{obs}$ characterize the density and total coverage of the obstacles. within the working area, respectively, and $S_{total}$ represents the total area of the entire working area. The proposed adaptive step-size is defined as:

(16) $$d_{step}=\{\begin{array}{ll} \frac{4L(1-B_{s})}{e^{B_{n}}(1+2n)}, & 0<n\leq 3\\ \frac{L}{2}, & n>3 \end{array}$$

Here, *n* denotes the count of obstacles located in the area extending across from the agricultural robot’s current position to the goal, while *L* represents the normalized expansion step size. The algorithm employs an adaptive step size strategy; specifically, in obstacle-free environments (*n* = 0), a maximum step size of 4*L* is utilized to enhance the algorithm’s exploration capability. As the environment becomes more densely populated and cluttered, the step size progressively diminishes. To ensure a balance between navigation efficiency and safety within cluttered regions, the step size is fixed at a minimum of 0.5*L* once the obstacle count surpasses the threshold of 3 (*n >* 3).

In Eq.16, we use *L* as the normalized step-length parameter. The *n* denotes the number of obstacles located in the region between the current node and the goal direction. Based on repeated experiments in our 100×100 maps, we treat n > 3 as a practical indicator of a crowded region; in such cases, large steps may overshoot narrow passages and reduce success rate. Therefore, when n > 3, we enforce a minimum step size to maintain stable exploration and collision-free expansion. In real scenarios, this threshold can be adjusted according to sensor range, map resolution, and crop-row spacing, or tuned online using local clearance measurements.

### 4.4 Path optimization

**Figure 6:**
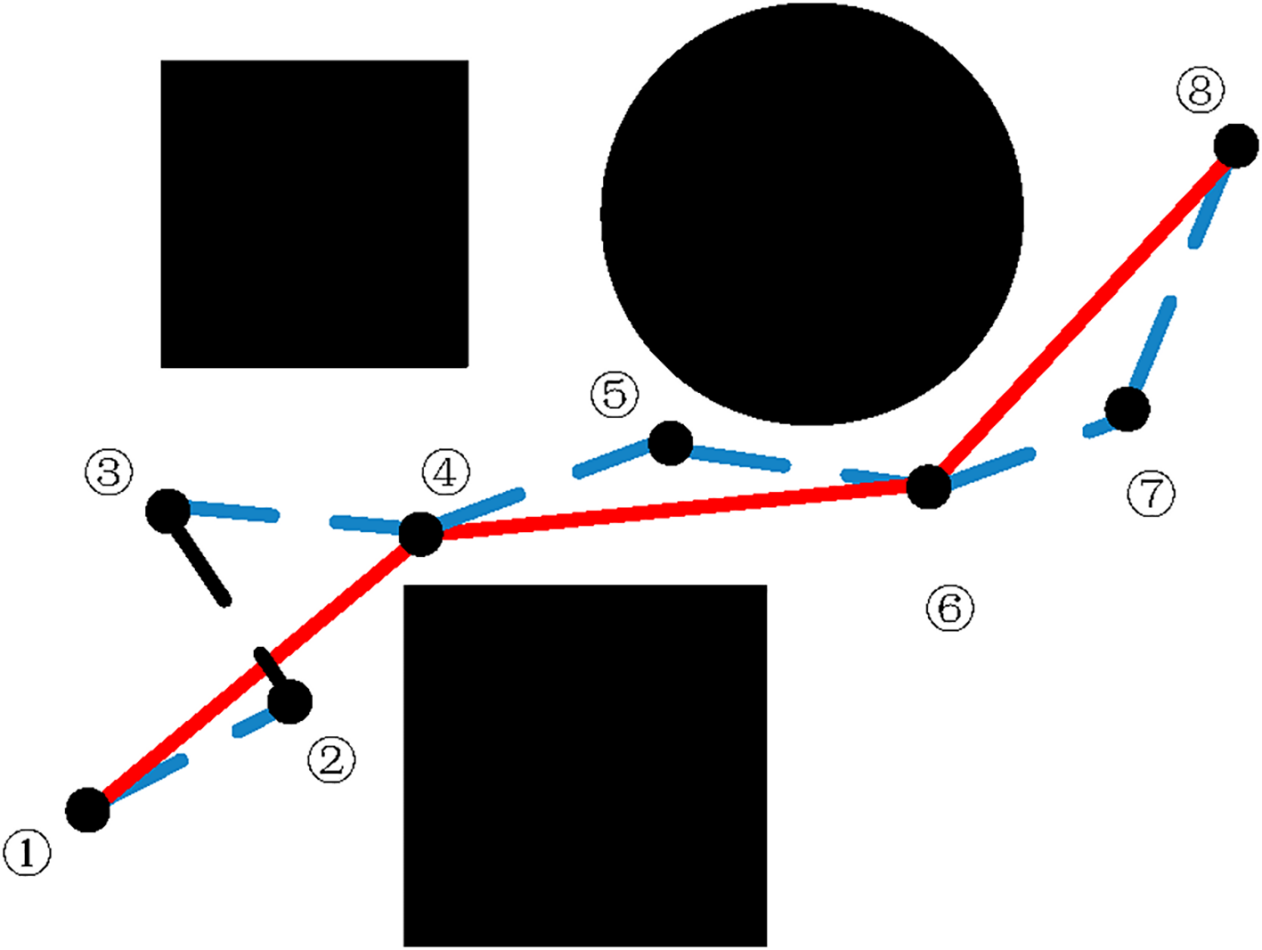
Schematic diagram of pruning principle.

In the preceding subsections, we focused on refining the internal structure of the algorithm, which significantly improved both the quality of the initial path and the search efficiency. However, the initial path generated retains a degree of inherent stochasticity, resulting in the presence of redundant nodes along the trajectory. While the existence of these redundant nodes inflates the path cost, it simultaneously indicates that further improvements in path quality are still possible. To tackle the problem of redundant nodes, this paper employs a trimming approach utilizing the triangle inequality principle. Upon generation of the initial path, this strategy initiates from the goal point and performs sequential collision checks against preceding nodes. If a collision-free connection exists, the nodes are linked directly. **Figure 6** illustrates the specific steps involved in this path simplification process.

While the primary objective of the triangle inequality pruning strategy proposed herein is the elimination of redundant nodes, its significance is notably amplified within the specific contexts of plant protection and agricultural logistics. Agricultural robots frequently operate on deformable terrains—such as soft soil or muddy greenhouse floors—where wheel-terrain interactions are inherently complex. Frequent sharp turns and steering maneuvers that fail to adhere to kinematic constraints can compromise the stability of data transmitted by monitoring sensors. By mitigating node redundancy, the algorithm minimizes the number of required steering maneuvers. This reduction is critical for sensors engaged in monitoring tasks, as it directly enhances the stability of the transmitted image data.

1. Starting from the initial node, number each path node in sequence and record the node number information.
2. Starting from the first node, attempt to connect with each subsequent node. If the path between two nodes is feasible and satisfies the agricultural robot steering constraint, record the serial numbers of the connectable nodes.
3. Sort all the nodes that the current node can connect to in ascending order of their serial numbers, select the node with the largest serial number for connection, and generate the path.
4. This process is iterated, with the newly connected node serving as the starting point for each subsequent expansion, until the destination is achieved. The sequence of connected nodes from the origin to the target then constitutes the complete path.

After simplifying the initial path through this method, the path in **Figure 6** changes from the initial path to ①→④→⑥→⑧. Superfluous nodes are eliminated, resulting in a reduced path length.

**Figure 7:**
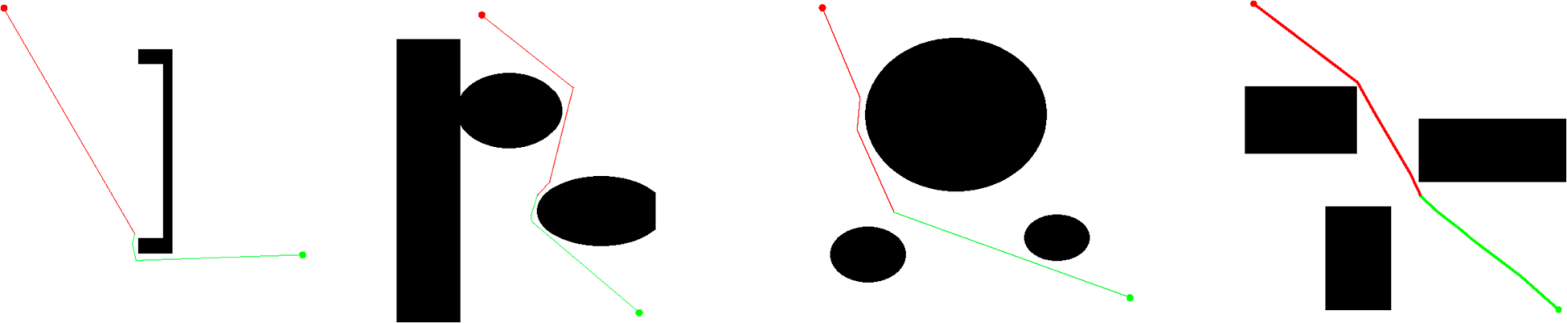
GABi-RRT* algorithm path map after pruning.

**Figure 7** shows a schematic diagram of the pruning treatment effect in environments with different obstacle distributions. This approach effectively reduces the path length while eliminating unnecessary nodes. However, the simplified path is still composed of segmented straight lines, and the smoothness at the turning points has not yet met the actual autonomous navigation in fields requirements. However, the pruning strategy adopted in this section effectively simplifies the tree structure, laying the foundation for the subsequent smoothing processing of B-spline curves.

Note that the pruning path consists of several straight segments, resulting in a sudden change in the steering angle at the connecting nodes. This leads to the Z-shaped trajectory being unsuitable for the kinematic characteristics of unmanned agricultural robots and causes camera jitter, which degrades the quality of pest and disease images. Smooth paths are essential for capturing clear, blur-free data during continuous monitoring. In order to optimize the initial path and ensure its kinematic feasibility, this paper introduces the cubic B-spline interpolation method, which has a smooth contour and limited curvature. This procedure guarantees that the resulting path adheres to the agricultural robot’s kinematic constraints, ensuring physical realizability. The mathematical expression of the cubic B-spline curve is given in Equation 17.

(17) $$F(u)=\sum_{i=0}^{3}L_{i}B_{i,3}(u),\;i=0,1,2,3$$

where *F*(*u*) denotes a point on the curve, *L_i_* represents the control points, *u* is the parameter along the curve, *i* is the index associated with the basis function, and *B_i,_*_3_(*u*) signifies the cubic B-spline basis function expression.

(18) $$L_{i}B_{i,3}(u)=\frac{1}{3!}\sum^{3-i}\;{\left(-1\right)}^{r}(\begin{array}{c} 4\\ r \end{array}){\left(u+3-i-r\right)}^{3}$$

**Figure 8:**
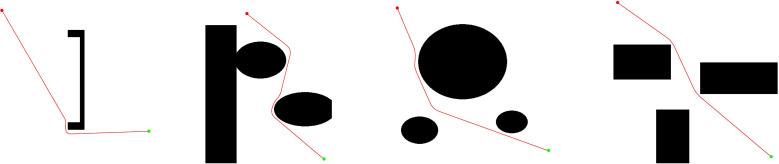
Diagram of the smoothing effect of the path.

**Figure 8** illustrates the comparative results of B-spline smoothing. It can be observed that the original path exhibits sharp geometric discontinuities at the turns, whereas the smoothed trajectory achieves continuous transitions in these areas. This optimization significantly enhances the continuity and drivability of the path for the robot.

However, since B-spline curves achieve this smoothness by approximating rather than interpolating control points, there is an inherent risk that the resulting trajectory may deviate from the safe corridor, particularly at sharp turns within narrow planting rows. To mitigate this risk, a post-smoothing safety validation is strictly implemented. The generated B-spline trajectory is first discretized into high-resolution waypoints, each of which undergoes a collision test against the environmental map. In the event that any portion of the smoothed curve is detected to intersect with an obstacle, the smoothing for that specific segment is discarded. Consequently, the system reverts to the original linear segment from the pruned path—which is guaranteed to be collision-free—thereby ensuring the absolute safety of the final executable trajectory.

Finally, the time complexity of RRT*-based algorithms is primarily dominated by the nearest neighbor search, scaling as *O*(*N* log*N*) for *N* iterations. In the proposed GABi-RRT*, the integration of APF forces and adaptive probability sampling (Eqs. 1-16) involves fixed-operation algebraic computations within a local scope. Theoretically, this introduces only a constant time overhead *O*(1) per iteration, preserving the overall asymptotic complexity of *O*(*N* log*N*). Furthermore, the B-spline optimization is strictly a post-processing step applied to the generated path nodes *K*. Since *K* ≪ *N*, its linear complexity *O*(*K*) has a negligible impact on the total computational load. Consequently, while the computation per node increases marginally, the enhanced heuristic guidance significantly reduces the total number of iterations required for convergence, resulting in the net reduction in running time observed in the experiments.

## 5 Simulation results and data analysis

To validate its efficacy, the proposed GABi-RRT* algorithm is benchmarked against three algorithm: Bias-RRT*, Informed RRT*, and P-RRT*. All simulations were performed in MATLAB 2016b on a Windows 10–based desktop system, configured with an Intel^®^ Core™ i5–7400 CPU @ 3.00GHz and 4 GB of RAM.

### 5.1 Environmental settings and parameter design

A continuous domain with dimensions of 100m × 100m was employed as the simulation environment to validate the efficacy of the proposed approach, with the aim of replicating the operational domain of the algorithm in real-world smart agriculture scenarios. In these maps, black regions denote obstacles such as dense crop rows and greenhouse structures, while white regions represent samplable spaces. Four distinct scenarios were employed to assess the navigation capability of the algorithm.

**Figure 9:**
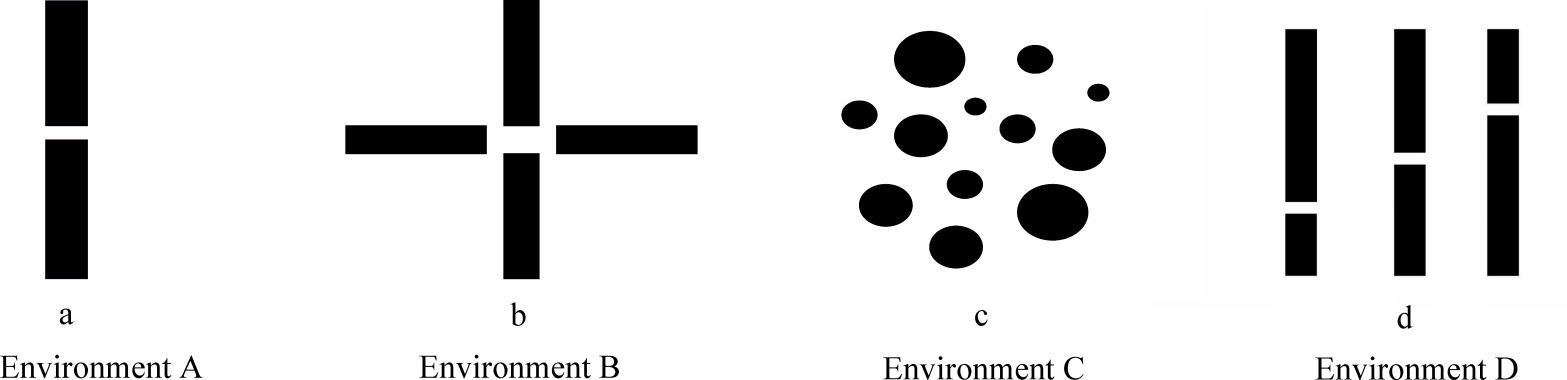
Simulation environments. **(a)** environment A, **(b)** environment B, **(c)** environment C, **(d)** environment D.

Environment A (**Figure 9a**) features a single narrow passage, simulating a high-density planting corridor where the lateral clearance is minimized. Environment B (**Figure 9b**) presents a narrow cross-passage topology, representing typical complex intersections and turning zones in greenhouse layouts. Environment C (**Figure 9c**) describes a dispersed multi-obstacle configuration, which includes twelve elliptical obstacles of varying sizes, simulating an unstructured area cluttered with irregular hazards such as rocks or irrigation equipment. Finally, Environment D (**Figure 9d**) illustrates a continuous narrow open environment, reflecting a multi-row crop monitoring path where the growth of different crop canopies creates alternating restricted and open regions. Moreover, we evaluate the algorithm based on the average path length of the first successful path found, the average running time, the stability across 50 independent trials, and the success rate.

In the experimental design, the start and target nodes of Environments A to D were initialized at coordinates (1, 1) and (99, 99), respectively. The Bias-RRT*, P-RRT*, and Bias-RRT* algorithms employed a fixed expansion step size of 1. This small-step strategy was implemented to accommodate the spatial constraints imposed by high-density planting environments, ensuring precise maneuvering within narrow passages. The GABi-RRT* algorithm adopted an adaptive step size strategy, obviating the need for predefined fixed step sizes. The target bias probability for both Bias-RRT* and GABi-RRT* was set to 0.3. For P-RRT* and GABi-RRT*, the attraction coefficient (*k_a_*) and repulsion coefficient (*k_p_*) were fixed at 1 and 10, respectively. All four algorithms were evaluated through 50 independent experiments under identical conditions across each of the four environmental types, with each experiment constrained to a maximum of 3000 sampling iterations. The detailed parameters are shown in **Table 1**.

**Table 1:** Parameter settings for the simulation environment and algorithms.

Category	Parameter	Symbol	Value
Simulation Params	Map Dimensions	$S_{total}$	100×100 m
	Start Configuration	$q_{start}$	(1,1)
	Goal Configuration	$q_{goal}$	(99,99)
	Maximum Iterations	$Iter_{max}$	3000
	Independent Runs	−	50
Algorithm Params	Fixed Step Size (Benchmark)	ϵ	1.0 m
	Adaptive Step Size (GABi-RRT*)	$d_{step}$	[0.5,4.0] m
	Target Bias Probability	$P_{bias}$	0.3
	Attraction Coefficient	$k_{a}$	1.0
	Repulsion Coefficient	$k_{r}$	10.0
	Obstacle Count Threshold	n	3

### 5.2 Experimental result

#### 5.2.1 Environment A

**Figure 10:**
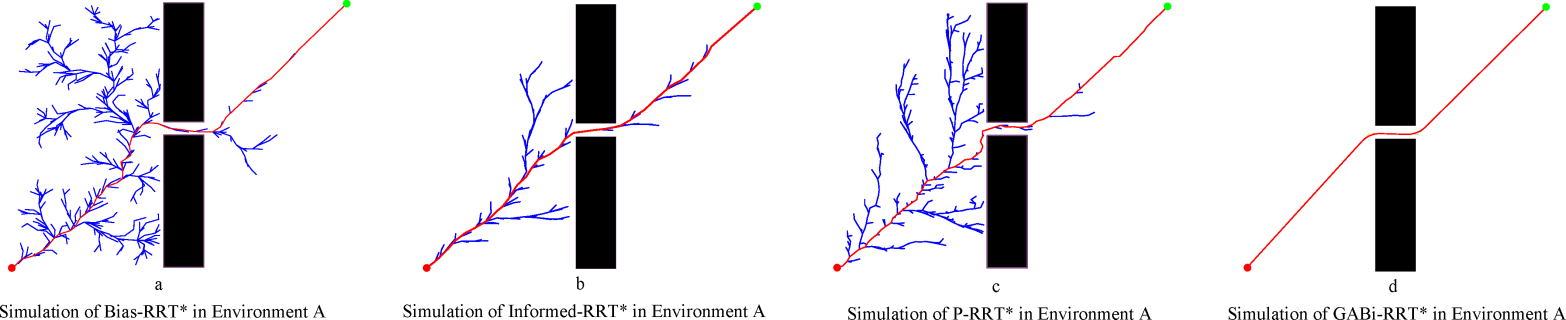
Simulation in environment A. **(a)** simluation of Bias-RRT* in evironment A, **(b)** simulation of Informed-RRT* in evironment A, **(c)** simulation of P-RRT* in environment A, **(d)** simluation of GABi-RRT* in environment A.

In Environment A, a comparative analysis of the four algorithms’ performance is presented in **Figure 10**. In generating optimal paths within environment A, where a clear performance disparity is evident. The Bias-RRT* algorithm, as depicted in **Figure 10a**, struggles with the narrow passages, resulting in trajectories of inferior quality and the lowest success rate. In contrast, the proposed GABi-RRT* algorithm (**Figure 10d**) demonstrates superior performance across all evaluated metrics. It not only achieves a 100% success rate but also yields the shortest average path length at 536.59 meters and exhibits the highest computational efficiency (lowest average run-time). Furthermore, the resulting path’s superior smoothness ensures better adherence to the agricultural robot’s kinematic constraints. In terms of computational efficiency, GABi-RRT* demonstrates a significant advantage. Specifically, the mean planning duration is decreased by 27.23%, 49.03%, and 70.99% compared to Bias-RRT*, Informed-RRT*, and P-RRT*, respectively. This substantial reduction indicates that the adaptive bidirectional search strategy effectively accelerates convergence in narrow single-passage scenarios.

**Figure 10** and **Table 2** confirm the superior path quality of the GABi-RRT* algorithm over the other three methods. Specifically, its average path length is reduced by 9.17%, 6.30%, and 2.71% compared to Bias-RRT*, Informed-RRT*, and P-RRT*, respectively.

**Table 2:** Simulation data in maximum number of iterations of 3000.

Environment	Algorithm	Successful rate	Average path length	Average time
Environment A	Bias-RRT*	90%	788.37	10.10
	Informed-RRT*	92%	772.23	14.42
	P-RRT*	78%	743.20	25.34
	GABi-RRT*	98%	726.59	7.35
Environment B	Bias-RRT*	98%	760.25	13.64
	Informed-RRT*	93%	751.38	18.25
	P-RRT*	85%	733.79	33.28
	GABi-RRT*	100%	714.68	7.64
Environment C	Bias-RRT*	90%	811.69	5.16
	Informed-RRT*	93%	806.54	7.12
	P-RRT*	100%	761.85	10.90
	GABi-RRT*	100%	730.72	2.99
Environment D	Bias-RRT*	92%	811.17	33.09
	Informed-RRT*	89%	796.76	35.93
	P-RRT*	72%	780.35	63.45
	GABi-RRT*	97%	765.29	18.07

#### 5.2.2 Environment B

**Figure 11:**
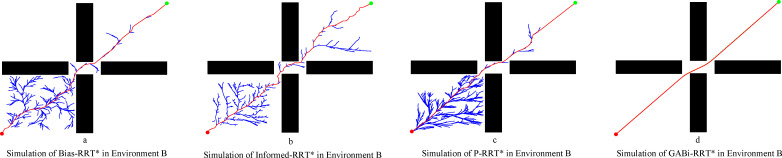
Simulation in environment B. **(a)** simluation of Bias-RRT* in evironment B, **(b)** simulation of Informed-RRT* in evironment B, **(c)** simulation of P-RRT* in environment B, **(d)** simluation of GABi-RRT* in environment B.

A comparison of the performance of the four algorithms in the more complex Environment B is presented in detail in **Figure 11** and **Table 2**. While the strategies of Bias-RRT* and Informed-RRT* yield shorter paths by optimizing the sampling space (**Figures 11a, b**), the P-RRT* algorithm struggles. Its repulsive force mechanism proves detrimental in this environment, degrading path quality, increasing execution time, and resulting in a path generation success rate of only 85%. In stark contrast, GABi-RRT* demonstrates superior overall performance, excelling in average path length, smoothness, and computation time, which underscores its effectiveness and robustness (**Figure 11d**).

Consistent with the findings in Environment A, the proposed GABi-RRT* algorithm maintains its advantage in path quality, yielding average paths that are 6.38%, 5.14%, and 2.67% shorter than those of Bias-RRT*, Informed RRT*, and P-RRT*, respectively. For the complex cross-passage topology in Environment B, GABi-RRT* maintains superior time efficiency. The simulation results show that the proposed algorithm reduces the average running time by 29.33% against Bias-RRT*, 47.18% against Informed-RRT*, and 71.03% against P-RRT*. This validates that the adaptive step-size strategy can quickly navigate through intricate intersections without excessive computational overhead.

#### 5.2.3 Environment C

**Figure 12:**
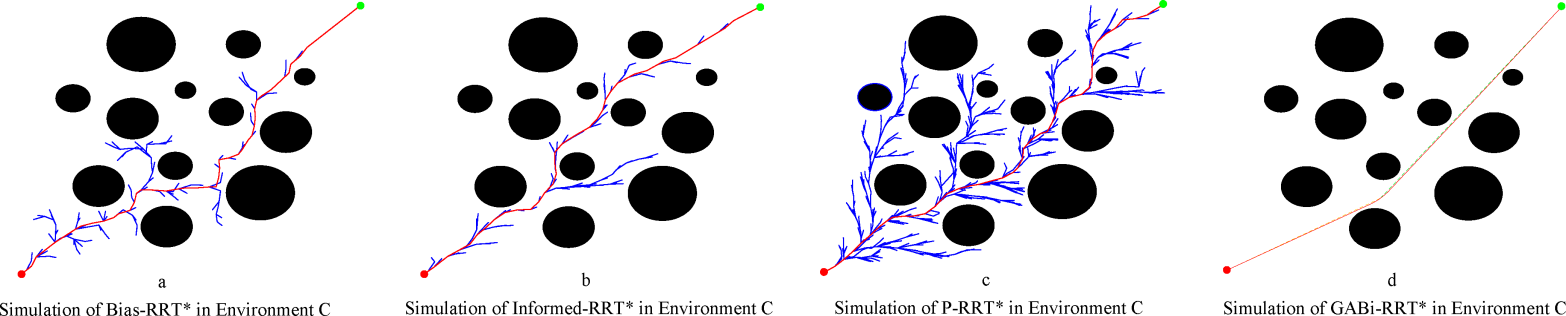
Simulation in environment C. **(a)** simluation of Bias-RRT* in evironment C, **(b)** simulation of Informed-RRT* in evironment C, **(c)** simulation of P-RRT* in environment C, **(d)** simluation of GABi-RRT* in environment C.

**Figure 12** illustrates the optimal paths generated within environment C, where the proposed GABi-RRT* algorithm again demonstrates its superiority. The path produced by our algorithm (**Figure 12d**) is qualitatively superior, exhibiting greater smoothness, fewer redundant nodes, and a shorter length. This visual assessment is quantitatively substantiated by the data in **Table 2**, which shows that GABi-RRT* reduces the average path length by 11.08%, 10.38%, and 4.26% compared to Bias-RRT*, Informed RRT*, and P-RRT*, respectively. While its 100% success rate in generating feasible paths is matched by P-RRT*, and the GABi-RRT* algorithm further highlights its rapid exploration capability. The planning time is shortened by 23.69%, 56.64%, and 73.01% compared to Bias-RRT*, Informed-RRT*, and P-RRT*, respectively. The efficient goal-biased sampling plays a crucial role in avoiding redundant searches in open spaces between obstacles. These findings together confirm the strong robustness and superior performance of GABi-RRT* in environments with dispersed obstacles.

#### 5.2.4 Environment D

**Figure 13:**
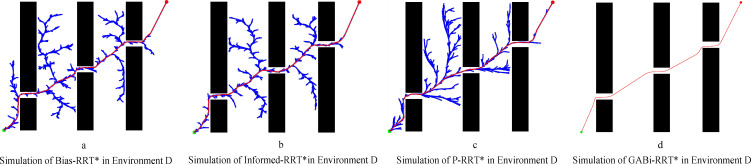
Simulation in environment D. **(a)** simluation of Bias-RRT* in evironment D, **(b)** simulation of Informed-RRT* in evironment D, **(c)** simulation of P-RRT* in environment D, **(d)** simluation of GABi-RRT* in environment D.

**Figure 13** evaluates the effectiveness of the four algorithms in Environment D, with quantitative metrics provided in **Table 2**. In the continuous narrow-open areas (**Figures 13a, b**), the path lengths produced by the goal-biased (Bias-RRT*) and elliptical sampling (Informed-RRT*) strategies are nearly identical. In contrast, the extended continuous obstacles (**Figure 13c**) cause the P-RRT* algorithm to exhibit slow initial convergence and unstable path quality.

The GABi-RRT* algorithm notably excels, delivering a path that is both the shortest in length and the highest in smoothness. Its average path length is 5.70%, 3.97%, and 1.60% shorter than that of the Bias-RRT*, Informed-RRT*, and P-RRT* algorithms, respectively. Navigating Environment D with its continuous narrow-open transitions proved challenging for traditional planners, yet GABi-RRT* exhibited remarkable robustness. It outperformed Bias-RRT*, Informed-RRT*, and P-RRT* by reducing the computation time by 45.39%, 49.71%, and 71.52%, respectively. This significant improvement is attributed to the synergistic effect of the improved APF and variable probability sampling, thereby avoiding the tree from becoming trapped in local minima close to extended obstacles.

**Figure 14:**
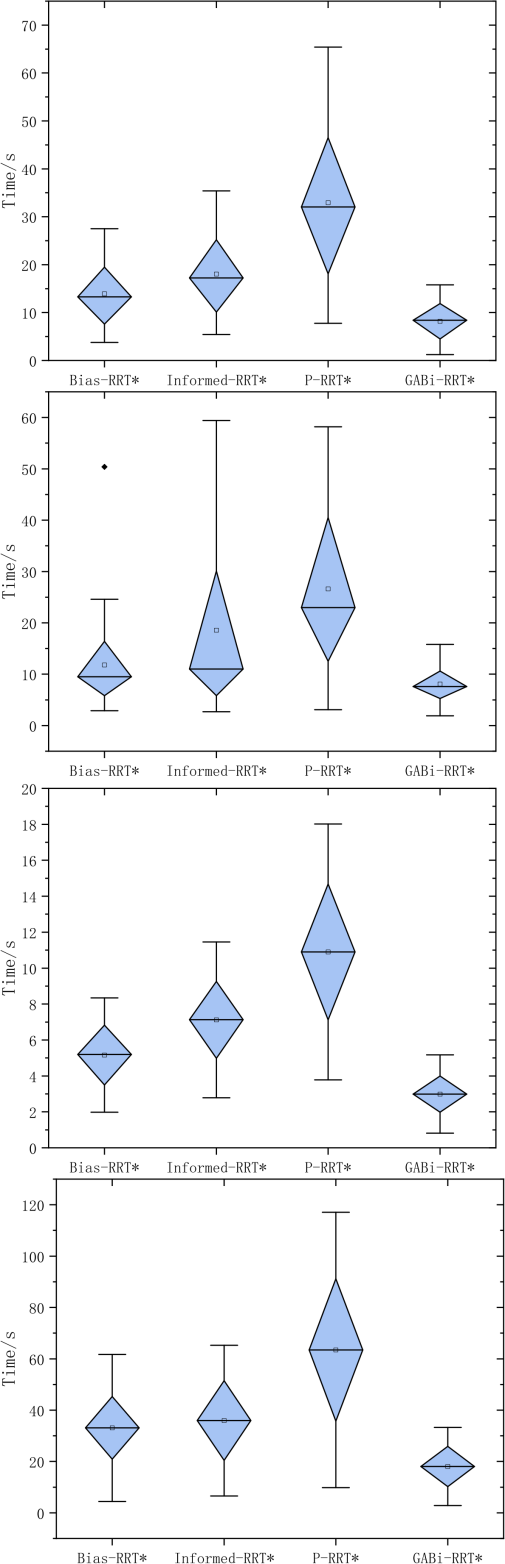
Box plot of algorithms running time of four environments.

To further evaluate the stability of the stochastic planners, we report box plots of the runtime over 50 independent trials for each environment (**Figure 14**). In Environment A (single narrow passage), the proposed GABi-RRT* exhibits the most concentrated distribution, with a smaller interquartile range (IQR) and fewer extreme values than Bias-RRT*, Informed-RRT*, and P-RRT*. This indicates that the adaptive sampling and step-size mechanisms reduce trial-to-trial fluctuation when the tree must repeatedly negotiate a narrow corridor. In Environment B (cross-passage topology), the variability of P-RRT* increases noticeably, which is consistent with its lower success rate and higher mean runtime; in contrast, GABi-RRT* maintains a compact IQR, suggesting more consistent convergence at intersections. In Environment C (dispersed multi-obstacle field), all methods benefit from the relatively open free space; nevertheless, GABi-RRT* still shows the tightest spread and the lowest median runtime, implying that the proposed goal-oriented sampling reduces redundant exploration between separated obstacles. The most challenging case is Environment D (continuous narrow–open transitions), where competing methods present wider boxes and more outliers, reflecting occasional stagnation and long runtimes in some trials. GABi-RRT* consistently yields a narrower IQR with fewer high-end outliers, demonstrating improved robustness against unfavorable random samples and reduced risk of excessive planning time. Overall, **Figure 14** confirms that the proposed method improves not only mean runtime but also runtime stability across diverse agricultural-like scenarios.

**Figure 15:**
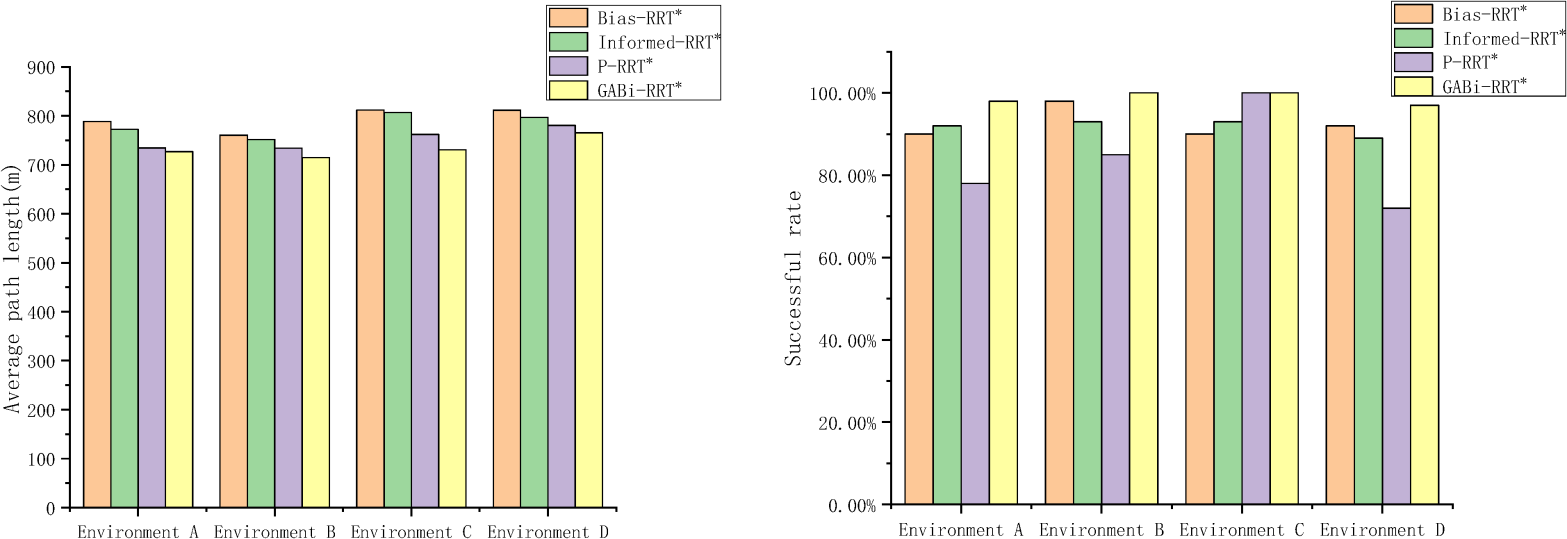
Successful rate and average path length.

The proposed GABi-RRT* algorithm demonstrates dual superiority in the three typical scenarios, achieving both the highest planning success rate and the shortest average path length after 3000 iterations. This performance advantage, which contributes to a lower overall path cost, is detailed in the comparative bar charts of **Figure 15**. These findings highlight the proposed scheme outperforms the classical RRT in terms of solution optimality, which can be primarily attributed to the enhanced adaptive attraction strategy and the adaptive sampling mechanism.

The proposed GABi-RRT* algorithm demonstrates dual superiority in the three typical scenarios, achieving both the highest planning success rate and the shortest average path length after 3000 iterations. This performance advantage, which contributes to a lower overall path cost, is detailed in the comparative bar charts of **Figure 15**. These findings highlight the proposed scheme outperforms the classical RRT in terms of solution optimality, which can be primarily attributed to the enhanced adaptive attraction strategy and the adaptive sampling mechanism.

## 6 Conclusion

This paper introduces the GABi-RRT* algorithm, designed to address the navigation challenges of autonomous mobile robots in precision pest and disease monitoring. To enhance global planning efficiency, a goal-oriented variable probability sampling strategy is implemented to provide dynamic guidance during node selection. This is complemented by an improved attraction mechanism based on the artificial potential field (APF) and an adaptive step-size strategy during the expansion stage, thereby substantially bolstering the robustness of the proposed framework within unstructured agricultural settings. Furthermore, a bidirectional search strategy is employed to accelerate the convergence rate. For trajectory optimization, GABi-RRT* integrates path pruning with cubic B-spline curve fitting. This dual approach not only eliminates redundant nodes but also ensures path smoothness—a critical factor for maintaining stable image acquisition and minimizing mechanical damage to crops during operation.

Simulation results validate that the proposed method yields substantial surpassing conventional methods in terms of computational speed and solution optimality, effectively solving the global planning problem in cluttered environments. However, it is acknowledged that the current validation is primarily conducted within simulation environments. Although these simulations rigorously model narrow and unstructured crop rows, real-world implementation involves physical uncertainties—such as wheel slip, sensor noise, and hardware computation constraints—that require further practical verification. Consequently, future research will focus on two primary directions: first, integrating the proposed global planner with a local reactive obstacle avoidance algorithm to enhance adaptability in dynamic environments; and second, implementing the algorithm on a physical pest detection platform to evaluate its real-time performance and robustness under actual field conditions.

## Data Availability

The original contributions presented in the study are included in the article/supplementary material. Further inquiries can be directed to the corresponding author.
